# Copper–Silver Bimetallic Nanowire Arrays for Electrochemical Reduction of Carbon Dioxide

**DOI:** 10.3390/nano9020173

**Published:** 2019-01-30

**Authors:** Yuanxing Wang, Cailing Niu, Yachuan Zhu

**Affiliations:** Institute of Advanced Synthesis, School of Chemistry and Molecular Engineering, Jiangsu National Synergetic Innovation Center for Advanced Materials, Nanjing Tech University, Nanjing 211816, Jiangsu, China; hyncljr@163.com (C.N.); zyc_660@163.com (Y.Z.)

**Keywords:** CO_2_ reduction, Cu–Ag nanowires, bimetallic nanocatalysts, electrocatalysis

## Abstract

The electrochemical conversion of carbon dioxide (CO_2_) into gaseous or liquid fuels has the potential to store renewable energies and reduce carbon emissions. Here, we report a three-step synthesis using Cu–Ag bimetallic nanowire arrays as catalysts for electrochemical reduction of CO_2_. CuO/Cu_2_O nanowires were first grown by thermal oxidation of copper mesh in ambient air and then reduced by annealing in the presence of hydrogen to form Cu nanowires. Cu–Ag bimetallic nanowires were then produced via galvanic replacement between Cu nanowires and the Ag^+^ precursor. The Cu–Ag nanowires showed enhanced catalytic performance over Cu nanowires for electrochemical reduction of CO_2_, which could be ascribed to the incorporation of Ag into Cu nanowires leading to suppression of hydrogen evolution. Our work provides a method for tuning the selectivity of copper nanocatalysts for CO_2_ reduction by controlling their composition.

## 1. Introduction

Carbon dioxide (CO_2_) is one of the end products of fossil fuels combustion. The accelerated consumption of fossil fuels leads to accumulated CO_2_ concentration in the atmosphere, which is widely considered as one reason for negative environmental consequences [1,2]. Electrochemical reduction can reduce atmospheric CO_2_ concentration and convert CO_2_ to a variety of useful chemicals, leading to significant changes in the utilization of CO_2_ [3,4,5,6]. In particular, the electrochemical reduction of CO_2_ in fuels and value-added chemicals, a relatively clean method, provides a promising approach to this goal [7,8].

Though the electrochemical reduction of CO_2_ on bulk metal electrodes has been studied for decades [9], more recent efforts have focused on nanostructured metallic catalysts for their enhanced catalytic performance over bulk materials [10,11]. Some noble metals, such as Au and Ag, preferably promote the formation of CO [12,13,14,15], whereas Pd and Sn preferably form formate [16,17]. Among the commonly studied metals, Cu is a unique catalyst that reduces CO_2_ to produce a variety of hydrocarbon and oxygenate products [18,19]. However, there are still challenges in the application of CO_2_ reduction to hydrocarbons by copper catalysts, such as low energetic efficiency and poor selectivity of desired products [20,21].

More recently, controlling the composition of nanomaterials has become an efficient way to improve the catalytic activity and selectivity for electrochemical reduction of CO_2_ [22]. The bimetallic approach has shown notable results, as it can influence the d-band center of the active components [23]. For instance, Kim et al. found that the selectivity of products could be controlled by tuning the composition of gold–copper bimetallic nanoparticles for electrochemical reduction of CO_2_ [24] Cu nanowires (NWs), a one-dimensional material, have shown higher activity over polycrystalline Cu for electrochemical reduction of CO_2_ owing to their high surface areas [25]. Bimetallic NWs have attracted much attention for their enhanced performance over monometallic NWs for catalytic and electrical applications [26,27]. As Ag is known to be selective for the formation of CO and less expensive than Au [28], it is rational to introduce Ag into Cu NWs to improve their catalytic performance for electrochemical reduction of CO_2_.

In this study, we demonstrate the use of galvanic replacement to synthesize Cu–Ag bimetallic NW arrays as catalysts for electrochemical reduction of CO_2_. The Cu–Ag NWs were produced by galvanic replacement of Cu NWs with silver, and the starting Cu NWs were obtained by a two-step method involving thermal oxidation of copper mesh and thermal reduction of the grown copper oxide NWs in the presence of hydrogen. The Cu–Ag NWs exhibited a higher selectivity of hydrocarbons and oxygenates over Cu NWs for electrochemical reduction of CO_2_. The enhanced catalytic performance was attributed to the incorporation of Ag, which could suppress hydrogen evolution.

## 2. Experimental Section 

### 2.1. Chemicals and Materials

Copper mesh (99.99%, 100 mesh), and carbon dioxide (CO_2_, 99.999%) were used as received. Potassium bicarbonate (KHCO_3_, 99.99%) was purchased from Macklin. Ascorbic acid (99.7%) was purchased from Sinopharm Chemical Reagent (Shanghai, China). Silver nitrate (AgNO_3_, 99.9999%) was purchased from Sigma Aldrich (St. Louis, United States). Hydrochloric acid was purchased from Shanghai Lingfeng Chemical Reagent (Shanghai, China). Anion exchange membrane was purchased from Tokuyama (Tokyo, Japan). Unless otherwise noted, all chemicals were used as received.

### 2.2. Copper Nanowire Array Synthesis

CuO/Cu_2_O NWs were grown on the copper mesh (100 mesh) adapted by Jiang et al. [29]. A piece of Cu mesh was first washed with 1 mol/L HCl solution for ~30 s to remove the oxide layer. After rinsing with deionized water and drying under N_2_ flow, the Cu mesh was placed in a combustion boat and annealed in a muffle furnace at 600 °C for 4 hours. Thermally reduced copper nanowires were obtained by annealing the CuO/Cu_2_O NWs at 300 °C for 2 h in a flow of forming gas (5%H_2_/N_2_, 30 sccm).

### 2.3. Copper–Silver Nanowire Array Synthesis

The copper NW arrays were immersed in a 20 mL scintillation vial filled with 0.55 mol/L ascorbic acid first. The solution was stirred rapidly for 5 minutes. To produce Cu–Ag nanowire arrays, the copper nanowire arrays were immersed in a solution containing 0.5 mmol/L AgNO_3_, and the contents were stirred for 3 minutes. Cu–Ag mesh was produced by a similar method, except copper mesh was used as starting material.

### 2.4. Material Characterization

Scanning electron microscopy (SEM) images were taken on an FEI Quanta FEG 250 microscope operated at 30 kV. Energy-dispersive X-ray spectroscopy (EDXS) elemental analyses were conducted using an EDXS attachment to the JEOL JEM 2100F transmission electron microscope (TEM). Nickel TEM grids were used for elemental analysis. X-ray diffraction (XRD) patterns were obtained on a Bruker D8 Advance X-ray diffractometer equipped with a Cu Kα source (λ = 1.5406 Å).

### 2.5. Electrochemical Studies

All electrochemical experiments were carried out in a customized gas-tight three-electrode electrochemical H-cell and a CHI 660E potentiostat. A Pt plate and an Ag/AgCl were used as the counter and reference electrode, respectively. A solution of 0.1 mol/L KHCO_3_ was prepared with 18.2 MΩ deionized water and used as the electrolyte. The pH value was measured at 6.8 when the electrolyte was saturated with CO_2_. CO_2_ was delivered to the cathode compartment at 10 standard cubic centimeter per minute (sccm). Before each measurement, the electrochemical cell was allowed to purge CO_2_ for 30 minutes, for the CO_2_ saturation of the electrolyte. The anode and cathode compartment was separated with an anion exchange membrane. All the applied potentials were reported versus reversible hydrogen electrode (RHE) potentials using E (vs. RHE) = E (vs. Ag/AgCl) + 0.199 V + 0.059 V × pH with 85% iR drop correction.

The CO_2_ electrochemical reaction was performed at ambient temperature and pressure. The gaseous products produced during the reaction were vented into the gas-sampling loop of a gas chromatograph (GC) with CO_2_ flow and analyzed online by a GC instrument (GC2060, Shanghai Ruimin) approximately every 15 minutes. The faradaic efficiency (FE) of gaseous products were calculated from the equation:

FE (%) = (*zFvG*p_0_)/(RT_o_*i*) × 100%

where *z* is the theoretical number of e- exchanged to form the gaseous products, *v* (vol%) is gaseous products volume concentration in the exhaust gas from the electrochemical cell, *G* (mL/min) is the gas flow rate, *i* is the cell current, *F* = 96,485 C/mol, p_0_ = 1.01 × 105 Pa, R = 8.314 J/(mol K), and T_0_ = 273.15 K [30].

The liquid products formed during the reaction were analyzed offline by a nuclear magnetic resonance spectrometer (Bruker Avance 400M, Karlsruher, Germany). For analysis, the electrolyte was mixed with D_2_O to form 90%:10% volume ratio solution and a known concentration of dimethyl sulfoxide as an internal standard. The ^1^H spectrum was measured with a water suppression method. FE of liquid products was calculated according to the equation:

FE (%) = *znF*/*Q* × 100 %

where *z* is the theoretic number of e- exchanged to form the liquid products, *n* is the moles of products, *Q* is the total charges applied, and *F* = 96,485 C/mol [31].

## 3. Results and Discussion

**Figure 1:**
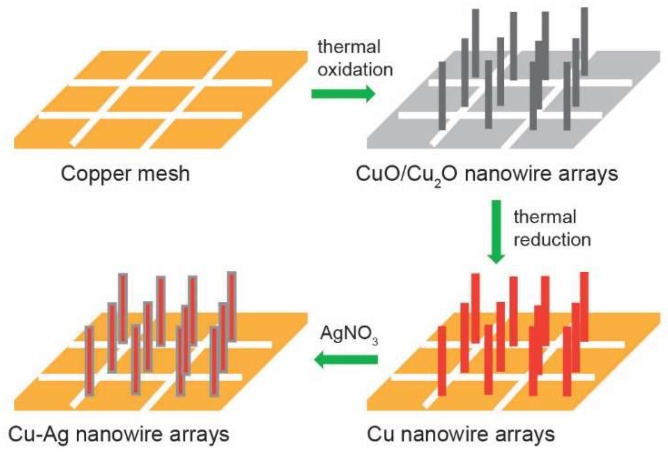
Schematic illustration of the synthesis of Cu–Ag nanowire (NW) arrays.

Figure 1 depicts the three-step synthesis process of Cu–Ag bimetallic NW arrays on the copper mesh. To prepare Cu NW arrays, a two-step method including thermal oxidation and reduction was adapted [25,29]. First, CuO/Cu_2_O NW arrays were synthesized by annealing copper mesh in ambient air. Then, the subsequent thermal reduction of CuO/Cu_2_O NW arrays was performed in a flow of forming gas to form Cu NW arrays. Last, the Cu NW arrays were immersed into an AgNO_3_ solution to yield Cu–Ag NW arrays for CO_2_ reduction. Detailed information about the synthesis can be found in the Experimental Section.

**Figure 2:**
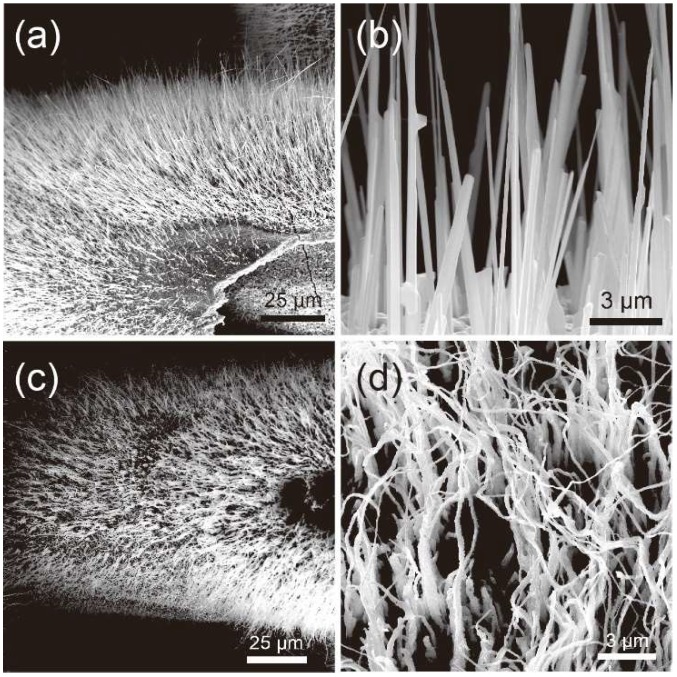
Low- and high-magnification SEM images of (**a**,**b**) as-synthesized CuO/Cu_2_O NWs and (**c**,**d**) thermally reduced Cu NWs.

Figure 2a,b shows the scanning electron microscope (SEM) images of synthesized CuO/Cu_2_O NW arrays. The CuO/Cu_2_O NWs were mainly grown vertically to the surface of the Cu mesh. Typical diameters of the produced CuO/Cu_2_O NWs were in the range of 50–100 nm with lengths up to 30 μm. The SEM images of the thermally reduced Cu NWs are shown in Figure 2c,d. Based on these images, the thermally reduced Cu NWs generally maintained the dimensions of the CuO/Cu_2_O NWs, despite some deformation of the head of the NWs. Additional SEM images can be found in the Supporting Information.

**Figure 3:**
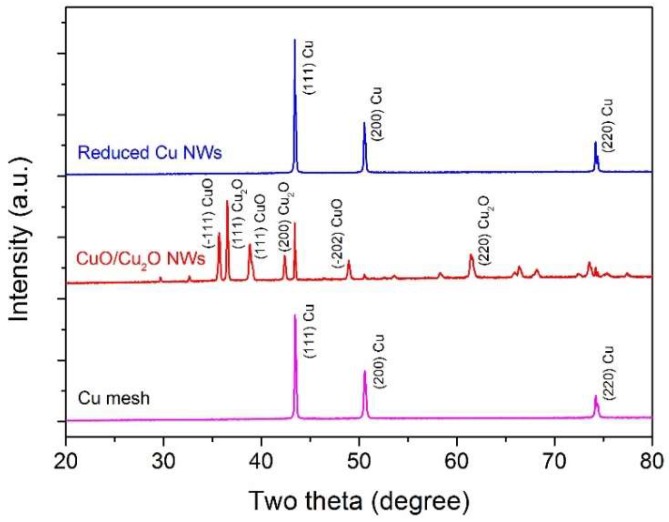
X-ray diffraction patterns of pristine copper mesh (magenta), annealed CuO NWs (red), and thermally reduced Cu NWs (blue).

To verify the crystal structure of the Cu mesh, annealed CuO/Cu_2_O NWs, and reduced Cu NWs, powder X-ray diffraction (XRD) measurements were made on representative samples. Figure 3 illustrates the XRD patterns of Cu mesh after annealing and reveals characteristic CuO reflections at 32.6, 35.6, 38.8, 48.9, 53.6, 58.3, 61.6, 66.3, 68.2, 72.5, and 75.4° (Joint Committee on Powder Diffraction Standards (JCPDS) card No. 05-0661), Cu_2_O reflections at 29.7, 36.5, 42.4, 73.6, and 77.4° (JCPDS card No. 65-3288), and metallic Cu reflections at 43.4, 50.6, and 74.3° (JCPDS card No. 65-9473). XRD pattern collected from reduced Cu NWs shows peaks of cubic Cu same as pristine Cu mesh. The XRD data indicate that annealing copper mesh in air produced high density CuO/Cu_2_O NWs, while the subsequent thermal reduction actually made cubic Cu NWs on the copper mesh. Additional details about these SEM and XRD measurements can be found in the Experimental Section.

**Figure 4:**
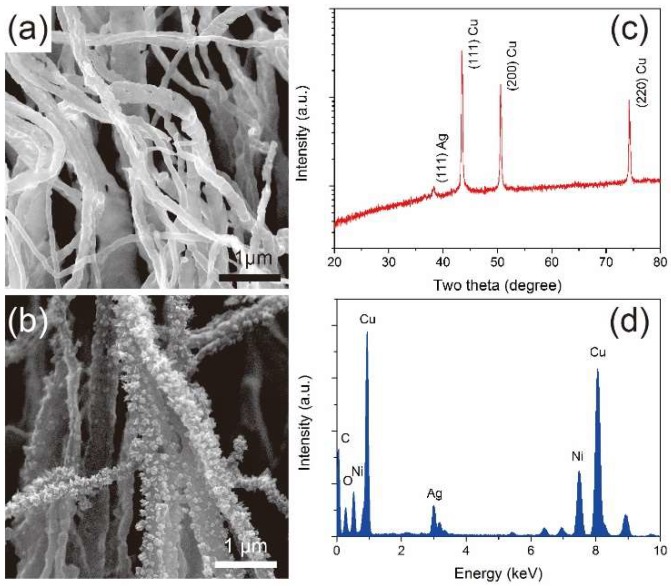
High-magnification SEM images of (**a**) thermally reduced Cu NWs, and (**b**) as-synthesized Cu–Ag NWs; (**c**) XRD pattern of the Cu–Ag NWs; (**d**) EDXS spectrum of the Cu–Ag NWs (Ni peak was from nickel TEM grid).

In recent years, galvanic replacement reactions have been applied as a synthetic tool for bimetallic nanomaterials [32,33]. By dipping Cu NWs into an AgNO_3_ solution, Cu–Ag NW arrays were obtained via galvanic replacement of silver according to the following equation [34,35]: Cu_(s)_ + 2Ag^+^ → Cu^2+^ + 2Ag_(s)_. Figure 4a,b shows the representative high-magnification SEM images of starting Cu NWs and obtained Cu–Ag NW arrays, respectively. The morphologies of these Cu–Ag NWs were similar to the Cu NWs (i.e., diameter of ~100 nm with length of up to 30 μm), despite some deposition on the surface of the Cu NWs. The rough surface could be ascribed to the large lattice mismatch between Cu and Ag [36]. To verify the crystal structure of Cu–Ag NWs, power XRD measurement was performed. The above metallic Cu diffraction peaks all appeared in the pattern of Cu–Ag NWs, but a new peak at 38.2° was consistent with (111) plane of cubic Ag (JCPDS No. 04-0783), suggesting some amount of Ag in the Cu–Ag NWs (Figure 4c). At this point, energy-dispersive X-ray spectroscopy (EDXS) measurement was carried out to determine the elemental information of the resulted bimetallic NWs. Based on Figure 4c, the Ag–Cu ratio was ~1:10 in the Cu–Ag bimetallic NWs. The EDXS mapping figure (Figure S3) indicated the uniform distribution of Cu and Ag on the NWs. The SEM, XRD, and EDXS data together indicated the successful synthesis of Cu–Ag bimetallic NWs. Detailed information about the synthesis can be found in the experimental section. Additional SEM images can be found in the supporting information.

Having established the synthesis of Cu–Ag NWs, electrochemical reduction of CO_2_ was conducted in CO_2_-saturated 0.1 mol/L KHCO_3_ electrolyte at ambient temperature and pressure. CO_2_ reduction experiments were performed in an electrolysis cell with the cathode and anode compartment separated by an anion exchange membrane, preventing the oxidation of CO_2_ reduction products. The cathodic compartment was purged with CO_2_ at a constant flow rate and vented into a gas chromatograph (GC) for quantification of gaseous products. The liquid products were analyzed by ^1^H nuclear magnetic resonance (NMR) spectroscopy after the completion of experiments.

**Figure 5:**
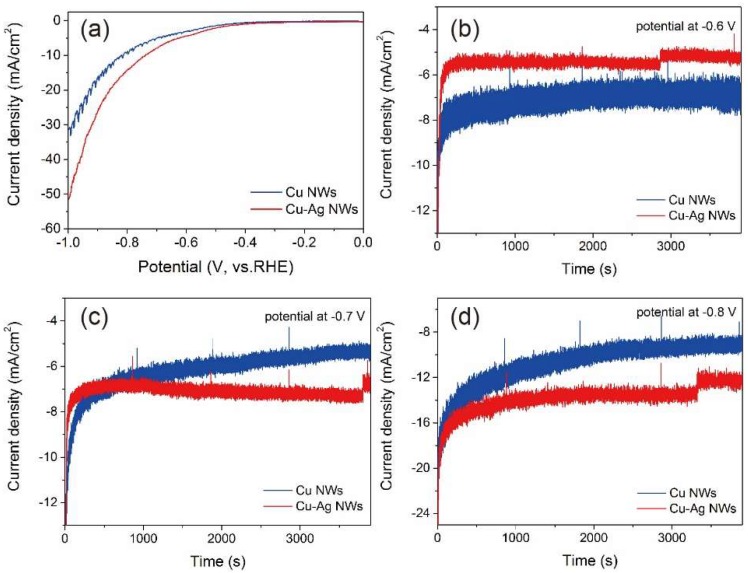
(**a**) Linear sweep voltammetry of electrochemical CO_2_ reduction on the Cu NWs and the Cu-Ag NWs with 5 mV/s scan rate. CO_2_ reduction activity on the Cu NWs and the Cu–Ag NWs at different potentials: (**b**) −0.6 V, (**c**) −0.7 V, and (**d**) −0.8 V.

The activity and selectivity of Cu NWs, Cu–Ag NWs, Cu mesh and Cu–Ag mesh for CO_2_ electroreduction were studied. Figure 5a shows the linear sweep voltammograms (LSV) of Cu NWs in comparison to the LSV of Cu–Ag NWs, using geometric current density as a comparison standard. Based on the LSV measurements, the Cu–Ag NWs generally exhibited a higher catalytic activity (larger negative current density and lower negative onset potential) over Cu NWs. To compare the long-term performance of each NW, their total geometric current density vs. time at various potentials are shown in Figure 5b–d, respectively. During the reaction, the decline in current densities for Cu NWs at various potentials indicates their unstableness for CO_2_ reduction. This phenomenon was more obvious at more negative potentials, such as –0.8 V vs. reversible hydrogen electrode (RHE; all potentials reported here are with respect to this reference). However, Cu–Ag NWs catalysts exhibited a steady-state current at constant potentials, which indicates the alloyed NWs were stable in the current electrochemical reaction conditions. The bimetallic NWs achieved a current density of ~6 mA/cm^2^ at relatively low overpotential (−0.6 V), which was even higher than the reported current density by using Ag NWs and Cu_2_O-derived films as catalysts [28,37]. The Cu–AG NWs showed higher catalytic activity for CO_2_ electroreduction over Cu NWs which could be ascribed to the addition of the Ag ingredient into Cu NWs, implying the impact of composition effects of Cu NWs catalysts for CO_2_ reduction. Additional details about the electrochemical studies can be found in the Experimental Section and Supporting Information.

**Figure 6:**
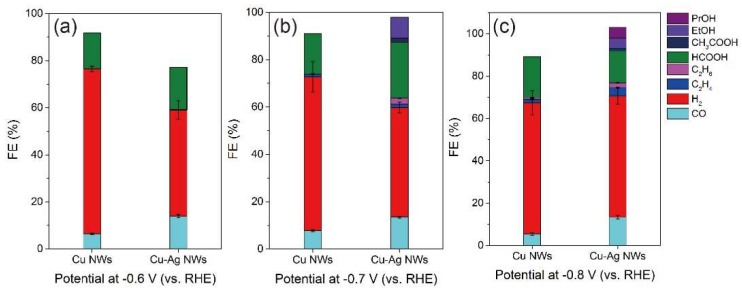
Faradaic efficiency (FE) of the major products for the Cu NWs and the Cu–Ag NWs at different applied potentials: −0.6 V (**a**), −0.7 V (**b**) and −0.8 V (**c**).

Figure 6 summarizes the faradaic efficiency (FE) of the desired products for the Cu NWs and Cu–Ag NWs at constant potentials during CO_2_ electroreduction. Figures S4–S6 summarize the selectivity of major products in the tests of long-term performance. For gaseous products, Ag–Cu NWs showed lower selectivity of H_2_ than the Cu NWs, along with higher selectivity of CO, C_2_H_4_, and C_2_H_2_ at all studied potentials. For liquid products, Ag–Cu NWs presented a higher selectivity of total liquid products over the Cu NWs. For example, the Cu NWs exhibited a H_2_ FE of 65%, a CO FE of 8%, and a formate FE of 17%, while the Cu–Ag NWs showed a H_2_ FE of 46%, a CO FE of 14%, and a formate FE of 24% at −0.7 V. In general, the Cu–Ag NWs exhibited enhanced catalytic selectivity of products towards CO_2_ electroreduction than that of the Cu NWs. The Cu–Ag NWs exhibited the best catalytic performance among all the studied materials (Figures S7–S12). It is important to note that the Cu NWs showed similar H_2_ selectivity as that of the Cu–Ag NWs at −0.8 V; this most likely reflects the mass transport limitations at high current density (e.g., >10 mA/cm^2^) rather than the intrinsic selectivity of these catalysts [18]. The enhancement in catalytic performance of the Cu–Ag NWs over Cu NWs may originate from different electronic structure by alloying a silver element into the copper composition [24]. Based on the density function theory (DFT) calculated binding energy of CO on the metal surface, Ag favors desorption of CO (an important reactive intermediate in the CO_2_ electroreduction) on its surface [38]. In addition, Ag adsorbs hydrogen weakly and the sluggish reaction–intermediate formation slows down the hydrogen evolution reaction [39]. The lower selectivity of H_2_, as well as higher selectivity of CO, hydrocarbons, and oxygenates after introducing Ag into Cu NWs, could be ascribed to the reasons mentioned above.

## 4. Conclusions

In summary, a simple three-step method was used to produce Cu–Ag NW arrays as electrocatalysts for CO_2_ reduction. CuO/Cu_2_O NWs were first grown by thermal oxidation of copper mesh, then thermal reduction was performed to form Cu NWs. By galvanic replacement with silver, Cu–Ag NWs were obtained while maintaining similar morphology and structure of the parent Cu NWs. The Cu–Ag NWs exhibited enhanced catalytic selectivity of hydrocarbons and oxygenates towards CO_2_ electroreduction over Cu NWs, attributed to the incorporation of Ag. Our work highlights the potential opportunity to tune the activity and selectivity of copper catalysts for CO_2_ electroreduction by controlling their composition.

## Supplementary Materials

The following are available online at http://www.mdpi.com/2079-4991/9/2/173/s1, Figure S1: High-magnification SEM images of as-synthesized CuO/Cu_2_O NWs. Figure S2: Low-magnification SEM images of as-synthesized Cu–Ag NWs. Figure S3: EDXS mapping figure of the Cu–Ag NWs (a) Cu distribution, (b) Ag distribution, and (c) TEM image. Figure S4: Time-dependent FE of major gaseous products for the Cu NWs and the Cu–Ag NWs at −0.6 V (vs. RHE). Figure S5: Time-dependent FE of major gaseous products for the Cu NWs and the Cu–Ag NWs at −0.7 V (vs. RHE). Figure S6: Time-dependent FE of major gaseous products for the Cu NWs and the Cu–Ag NWs at −0.8 V (vs. RHE). Figure S7: X-ray diffraction patterns of pristine copper mesh (blue) and Cu–Ag mesh (red). Figure S8: Linear sweep voltammetry of electrochemical CO_2_ reduction of the Cu mesh and the Cu–Ag mesh. Figure S9: CO_2_ reduction activity on the Cu mesh and the Cu–Ag mesh at different potentials. Figure S10: FE of the major products for the Cu mesh and the Cu–Ag mesh at −0.6 V (vs. RHE). Figure S11: FE of the major products for the Cu mesh and the Cu–Ag mesh at −0.7 V (vs. RHE). Figure S12: FE of the major products for the Cu mesh and the Cu–Ag mesh at −0.8 V (vs. RHE).
