# Supplementary material for: Copper–Silver Bimetallic Nanowire Arrays for Electrochemical Reduction of Carbon Dioxide
Source: Nanomaterials (Basel). 2019 Jan 30;9(2):173. doi: 10.3390/nano9020173 (PMC6410069; doi:10.3390/nano9020173)
Supplement: Supplementary file 1 [file nanomaterials-09-00173-s001.pdf]

Supporting Information

Copper-Silver Bimetallic Nanowire Arrays for Electrochemical Reduction of Carbon Dioxide

Yuanxing Wang[1,*], Cailing Niu[1] and Yachuan Zhu[1]

[1] Institute of Advanced Synthesis, School of Chemistry and Molecular Engineering, Jiangsu National Synergetic Innovation Center for Advanced Materials, Nanjing Tech University, Nanjing, Jiangsu 211816, P. R. China.

Correspondence to Y.W. (ias_yxwang@njtech.edu.cn)

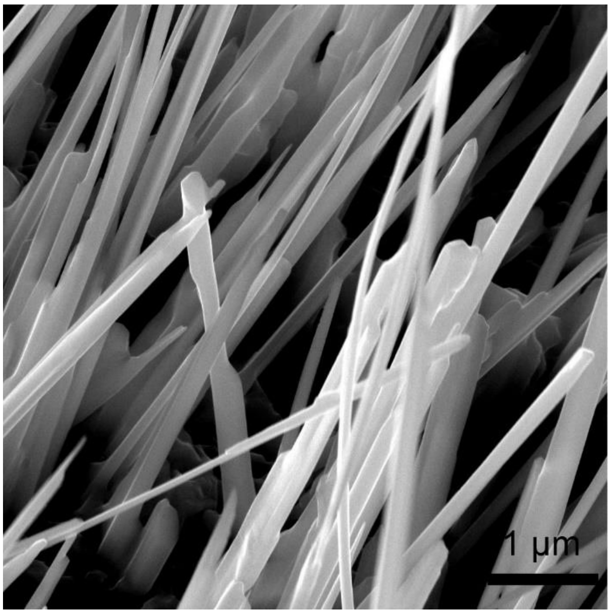

Figure S1. High-magnification SEM images of as-synthesized CuO/Cu_2O NWs.

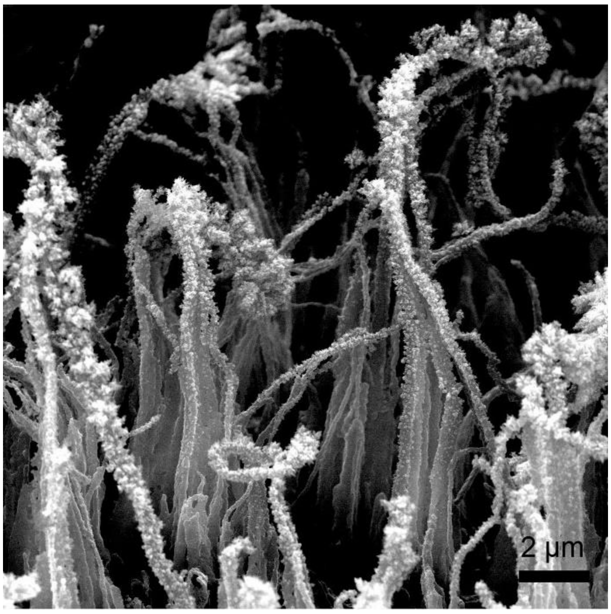

Figure S2. Low-magnification SEM images of as-synthesized Cu-Ag NWs.

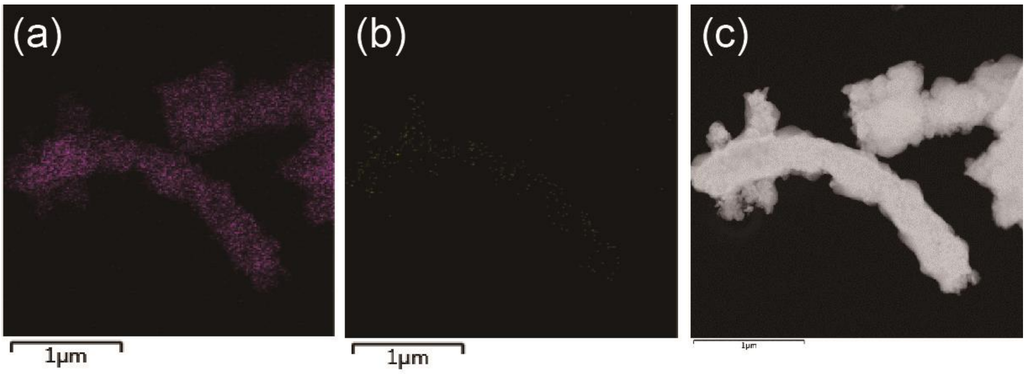

Figure S3. EDXS mapping figure of the Cu-Ag NWs (a) Cu distribution, (b) Ag distribution, and (c) TEM image.

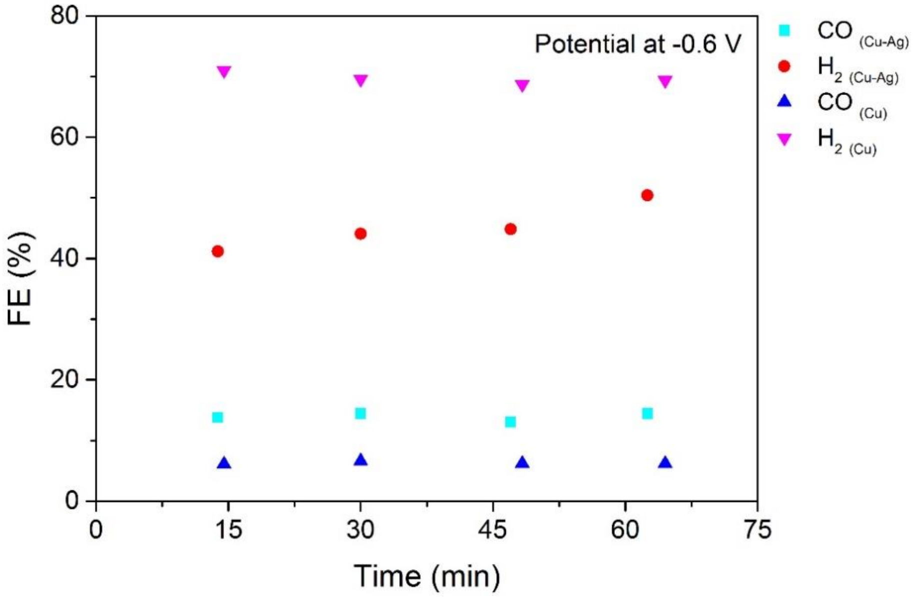

Figure S4. Time-dependent FE of major gaseous products for the Cu NWs and the Cu-Ag NWs at -0.6 V (vs. RHE).

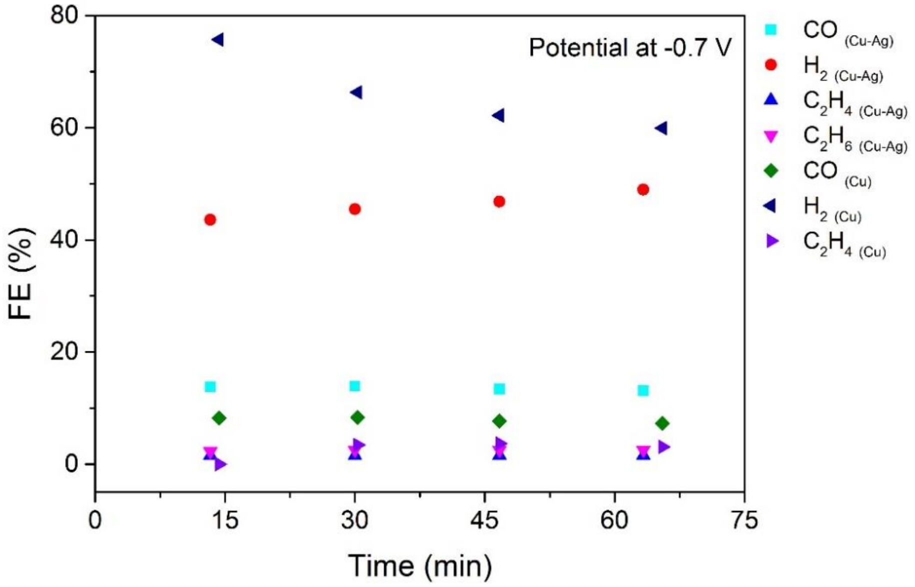

Figure S5. Time-dependent FE of major gaseous products for the Cu NWs and the Cu-Ag NWs at -0.7 V (vs. RHE).

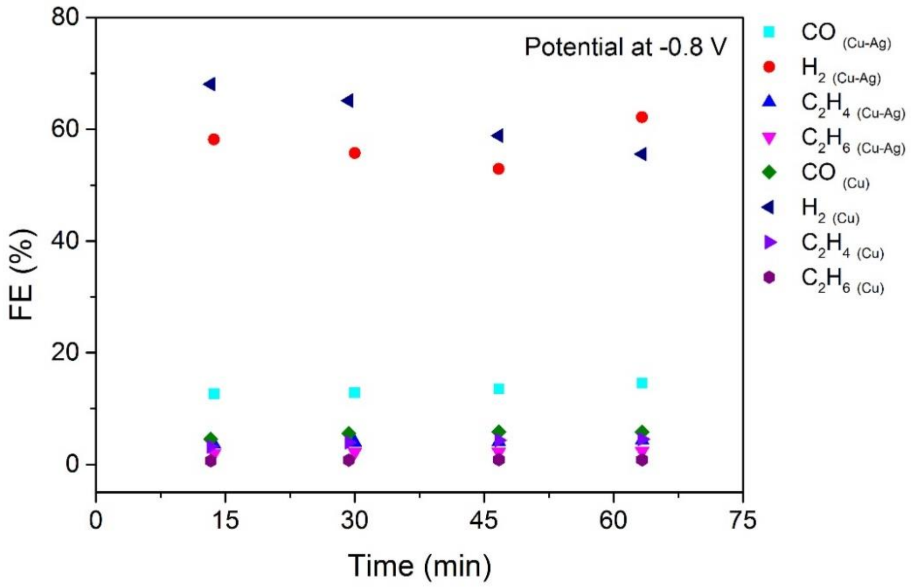

Figure S6. Time-dependent FE of major gaseous products for the Cu NWs and the Cu-Ag NWs at -0.8 V (vs. RHE).

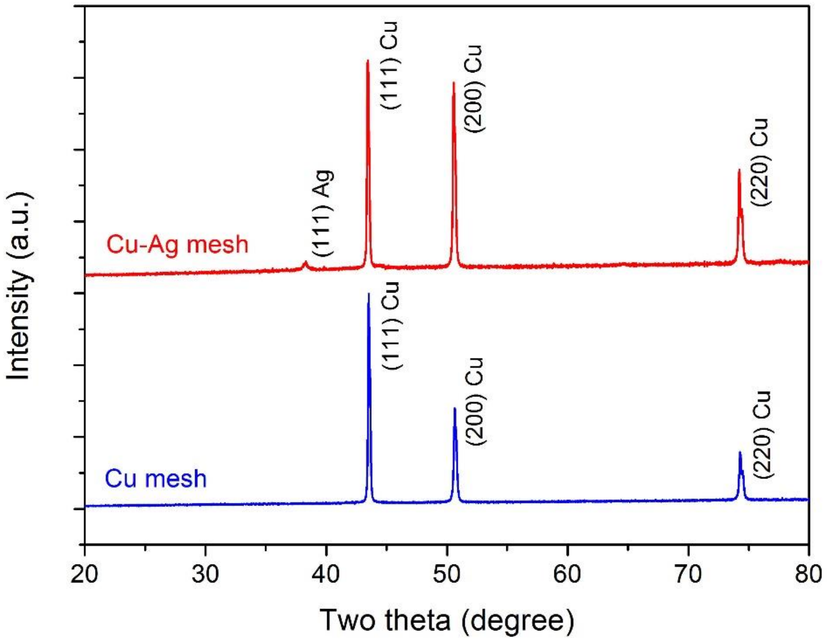

Figure S7. X-ray diffraction patterns of pristine copper mesh (blue) and Cu-Ag mesh (red).

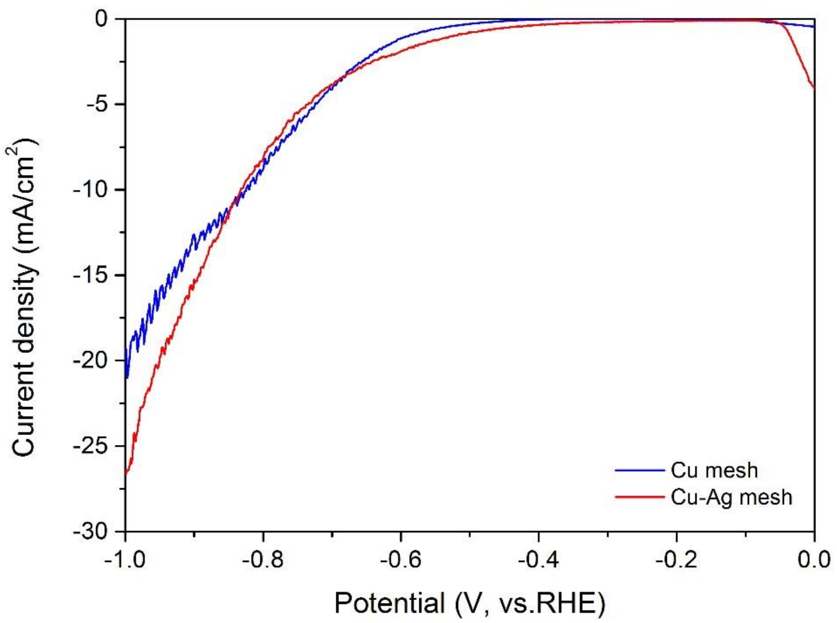

Figure S8. Linear sweep voltammetry of electrochemical CO_2 reduction of the Cu mesh and the Cu-Ag mesh.

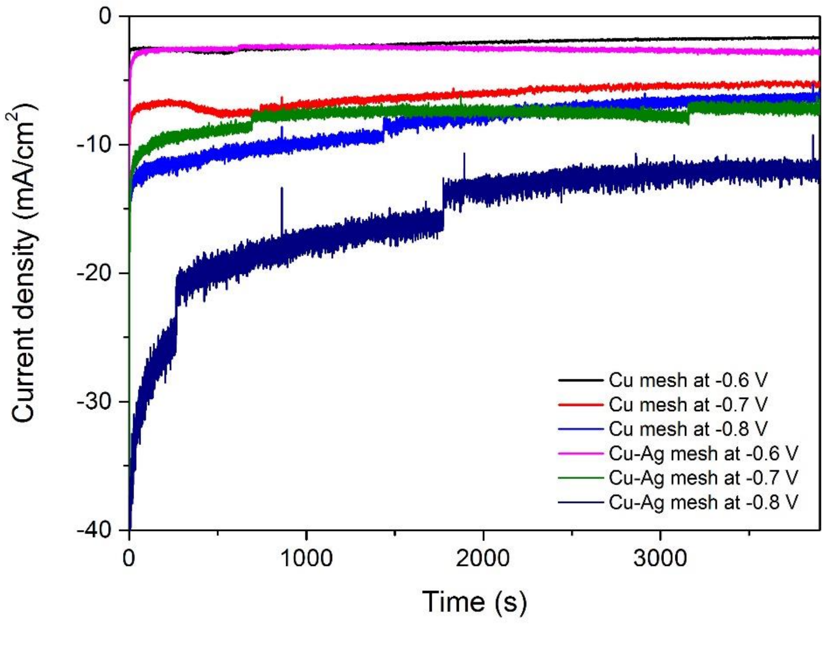

Figure S9. CO_2 reduction activity on the Cu mesh and the Cu-Ag mesh at different potentials.

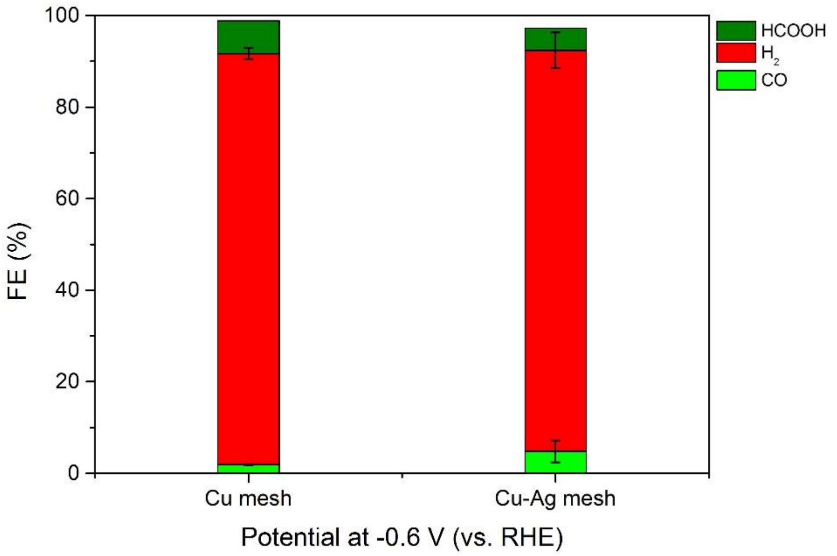

Figure S10. FE of the major products for the Cu mesh and the Cu-Ag mesh at -0.6 V (vs. RHE).

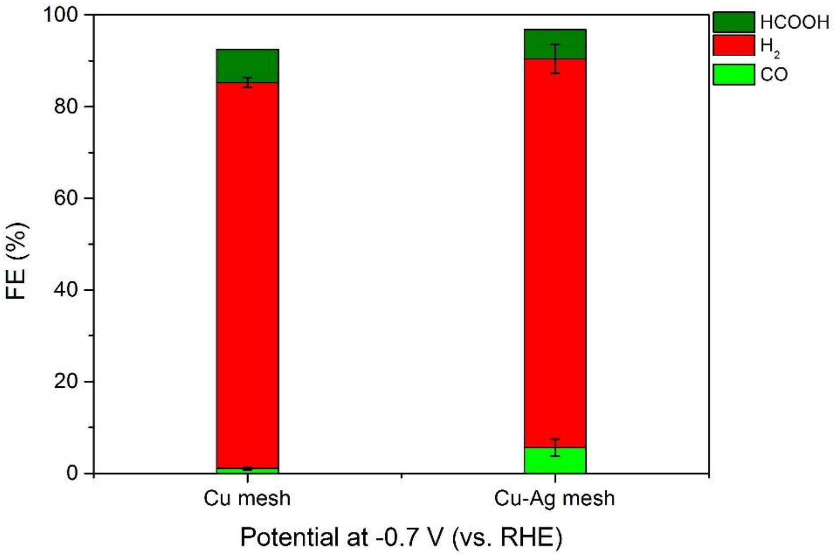

Figure S11. FE of the major products for the Cu mesh and the Cu-Ag mesh at -0.7 V (vs. RHE).

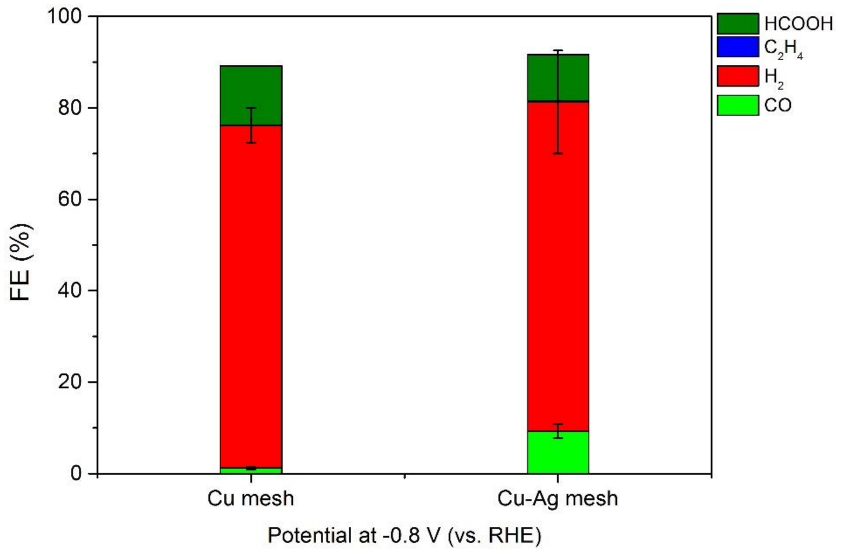

Figure S12. FE of the major products for the Cu mesh and the Cu-Ag mesh at -0.8 V (vs. RHE).
